# Supplementary figures and images for: Proteome Dynamics Analysis Reveals the Potential Mechanisms of Salinity and Drought Response during Seed Germination and Seedling Growth in Tamarix hispida
Source: Genes (Basel). 2023 Mar 5;14(3):656. doi: 10.3390/genes14030656 (PMC10048391; doi:10.3390/genes14030656)

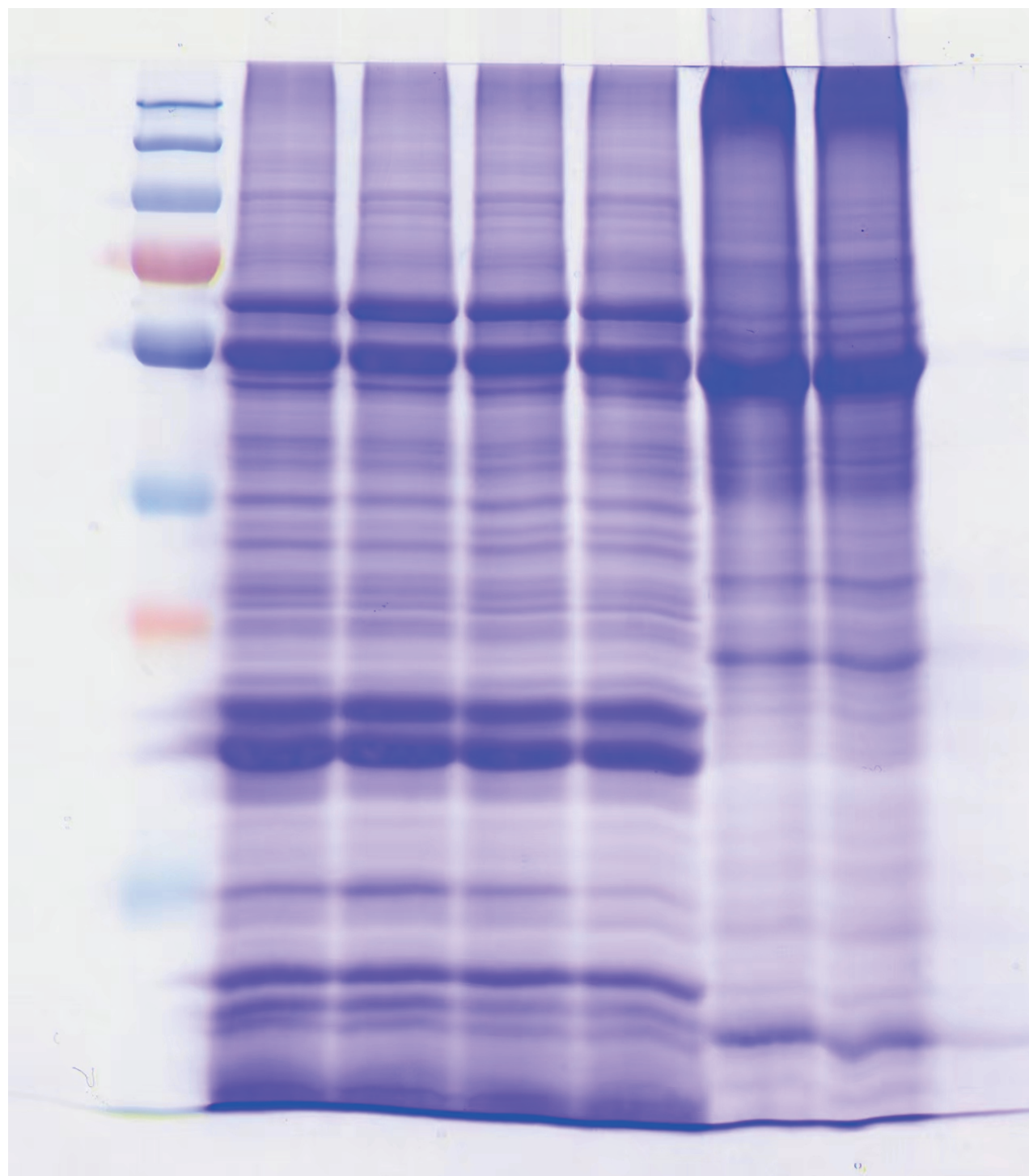

Supplement: Supplementary file 1 [file genes-14-00656-s001.zip › Figure S1.pdf]

a

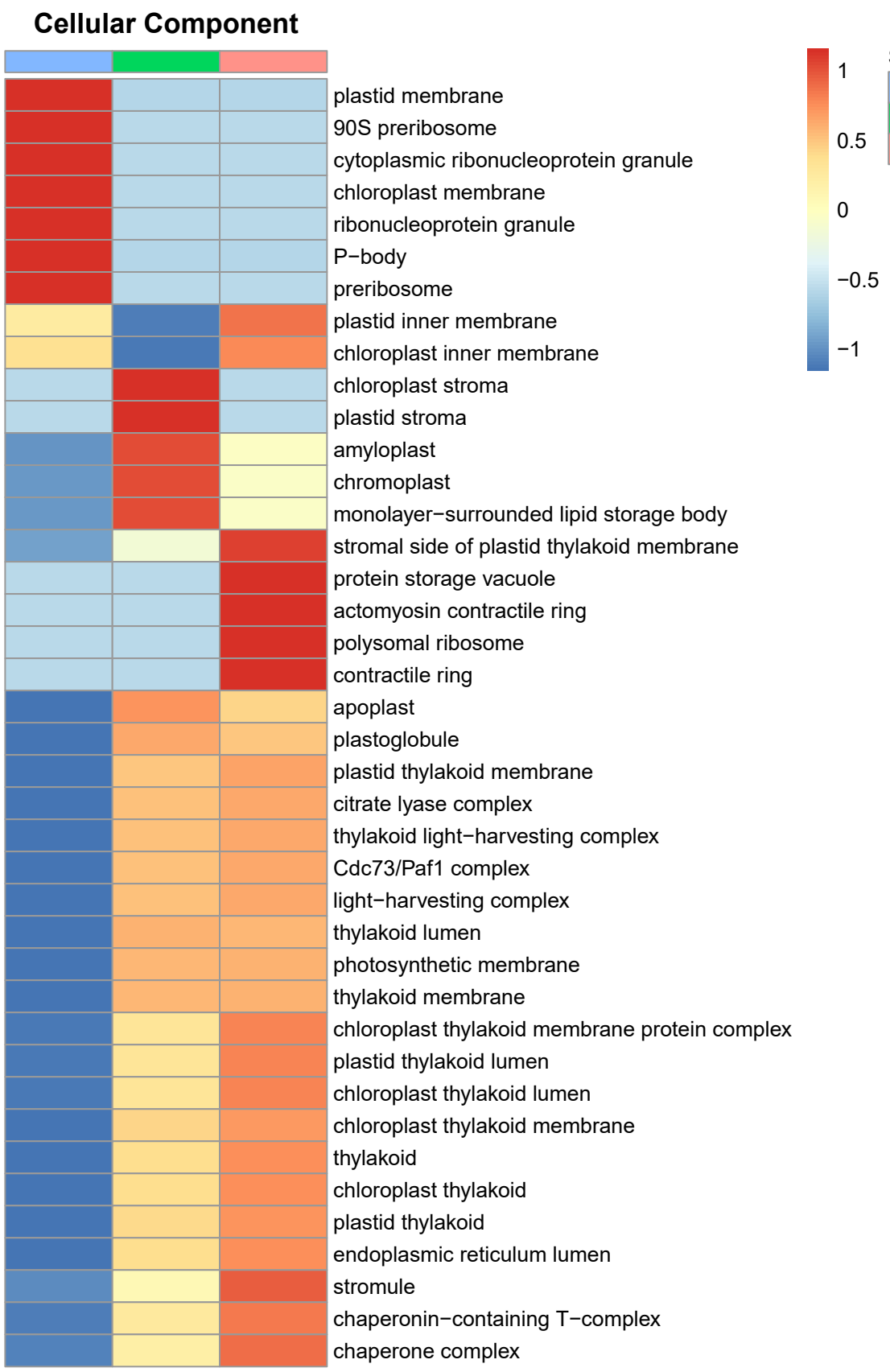

b

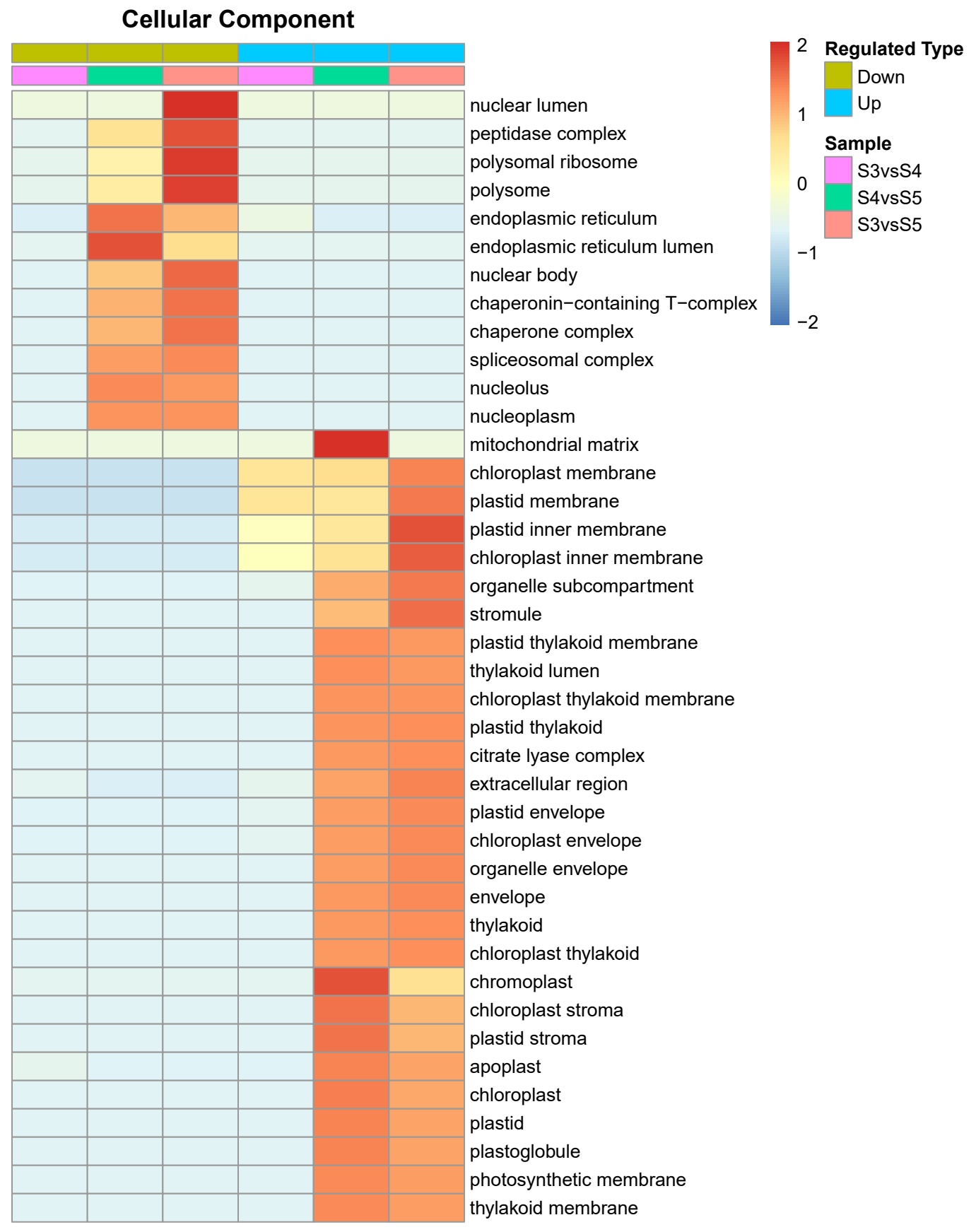

Supplement: Supplementary file 1 [file genes-14-00656-s001.zip › Figure S10.pdf]

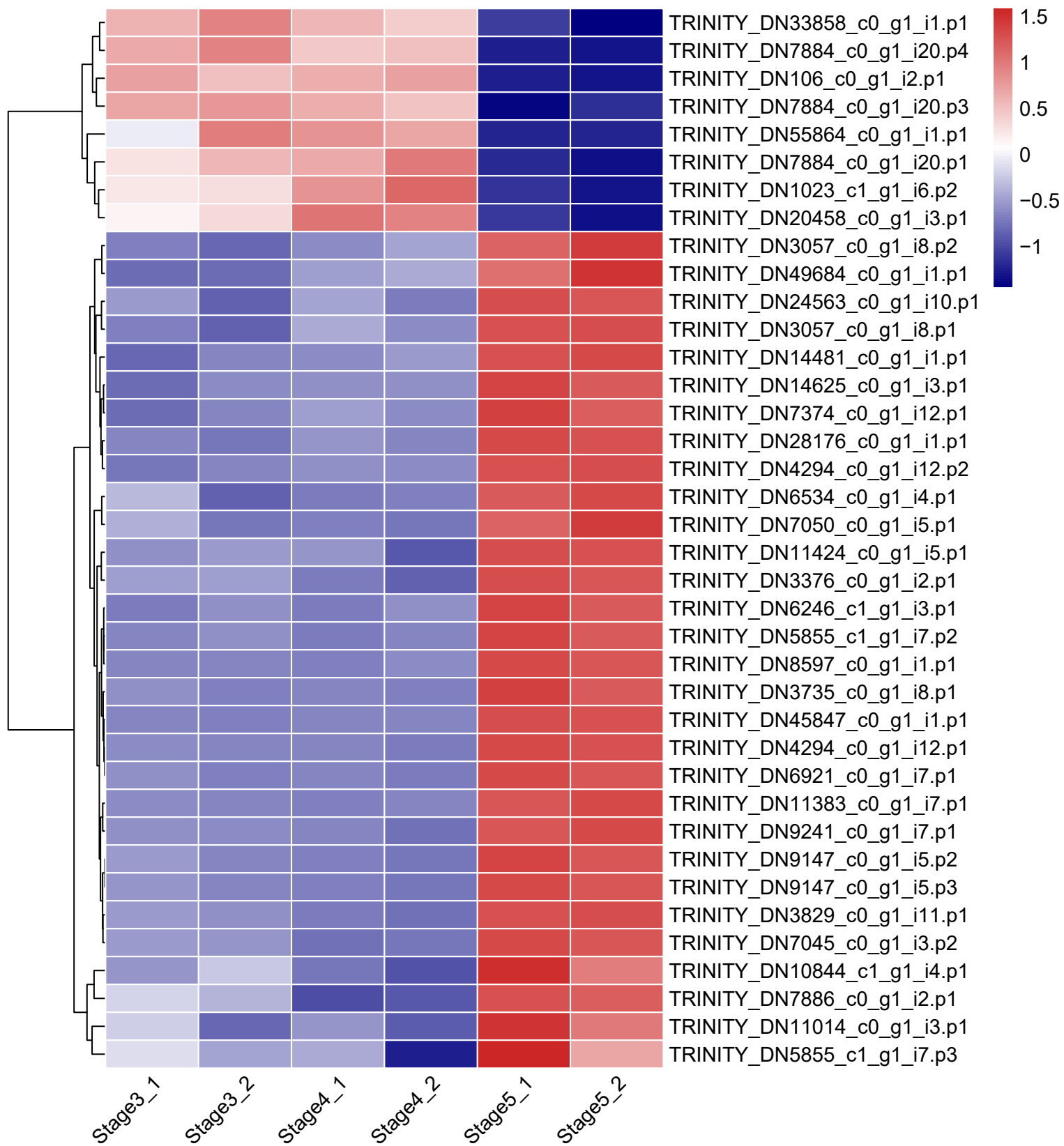

Supplement: Supplementary file 1 [file genes-14-00656-s001.zip › Figure S11.pdf]

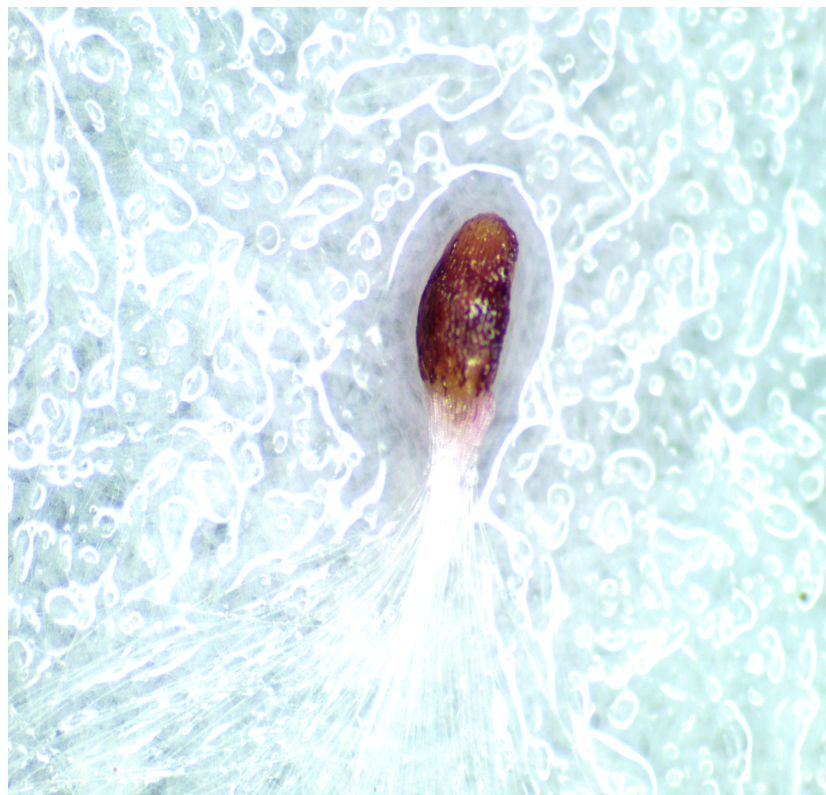

Stage 3

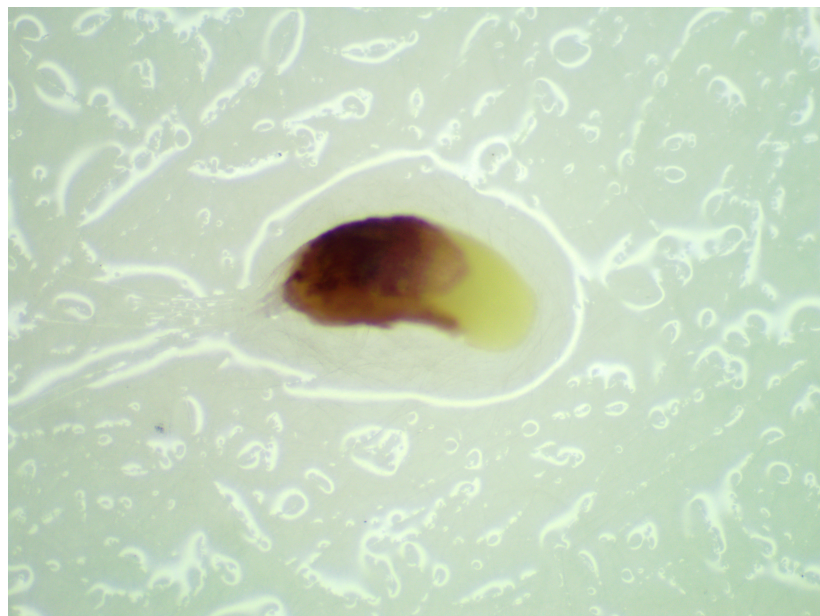

Stage 4

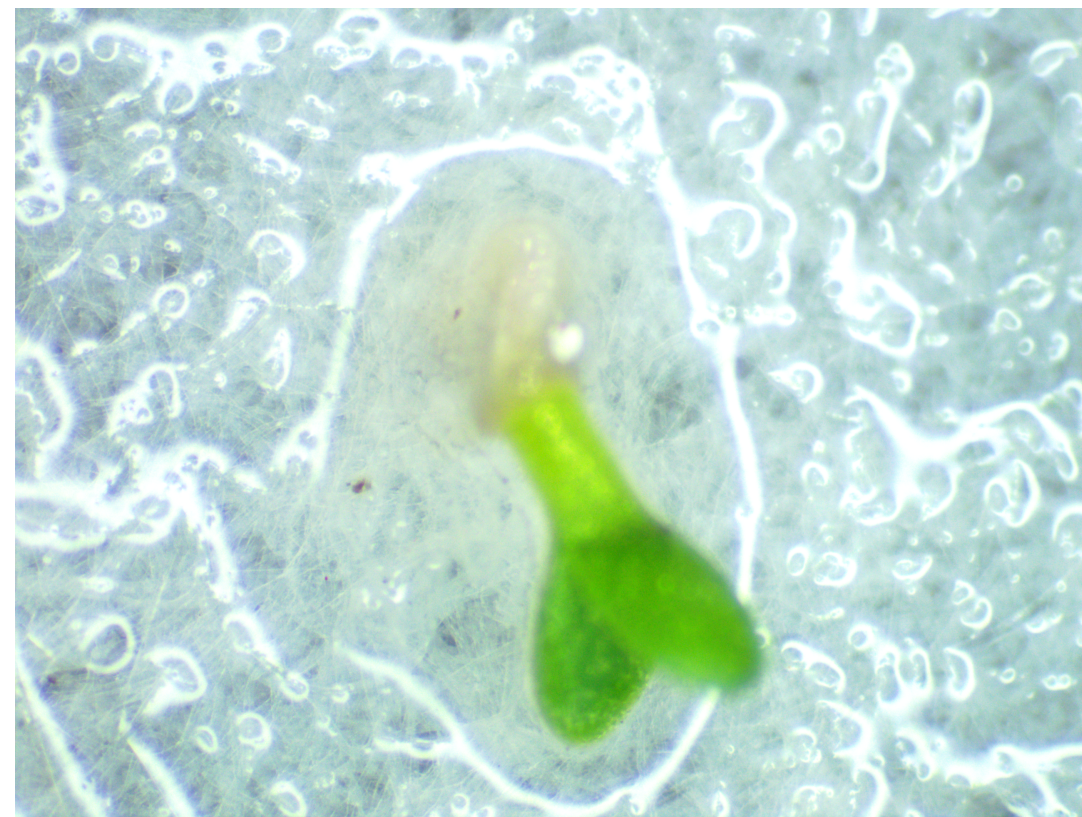

Stage 5

Supplement: Supplementary file 1 [file genes-14-00656-s001.zip › Figure S2.pdf]

a

## DEGs, Stage3 vs Stage4

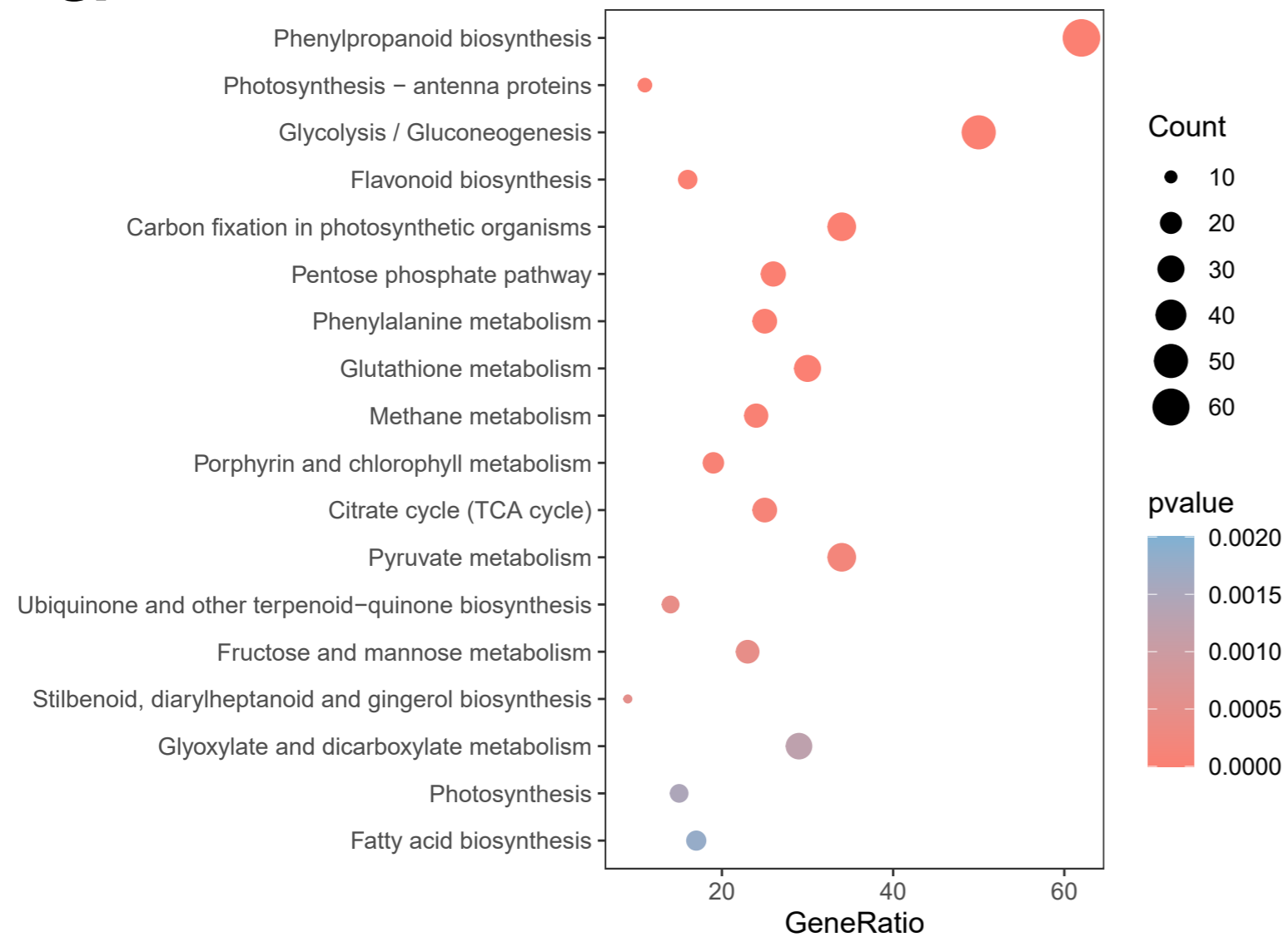

b

## DEGs, Stage4 vs Stage5

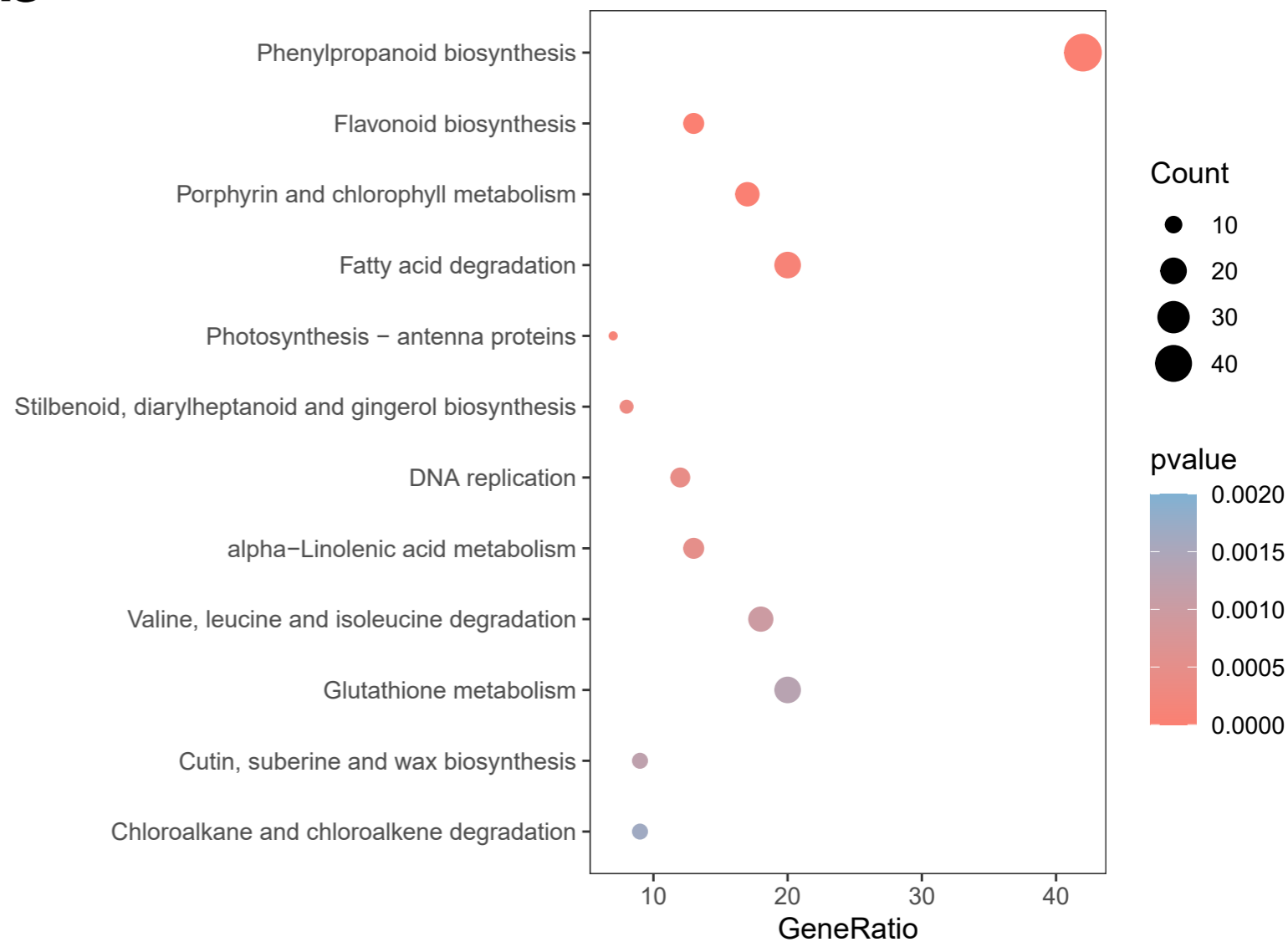

Supplement: Supplementary file 1 [file genes-14-00656-s001.zip › Figure S3.pdf]

a

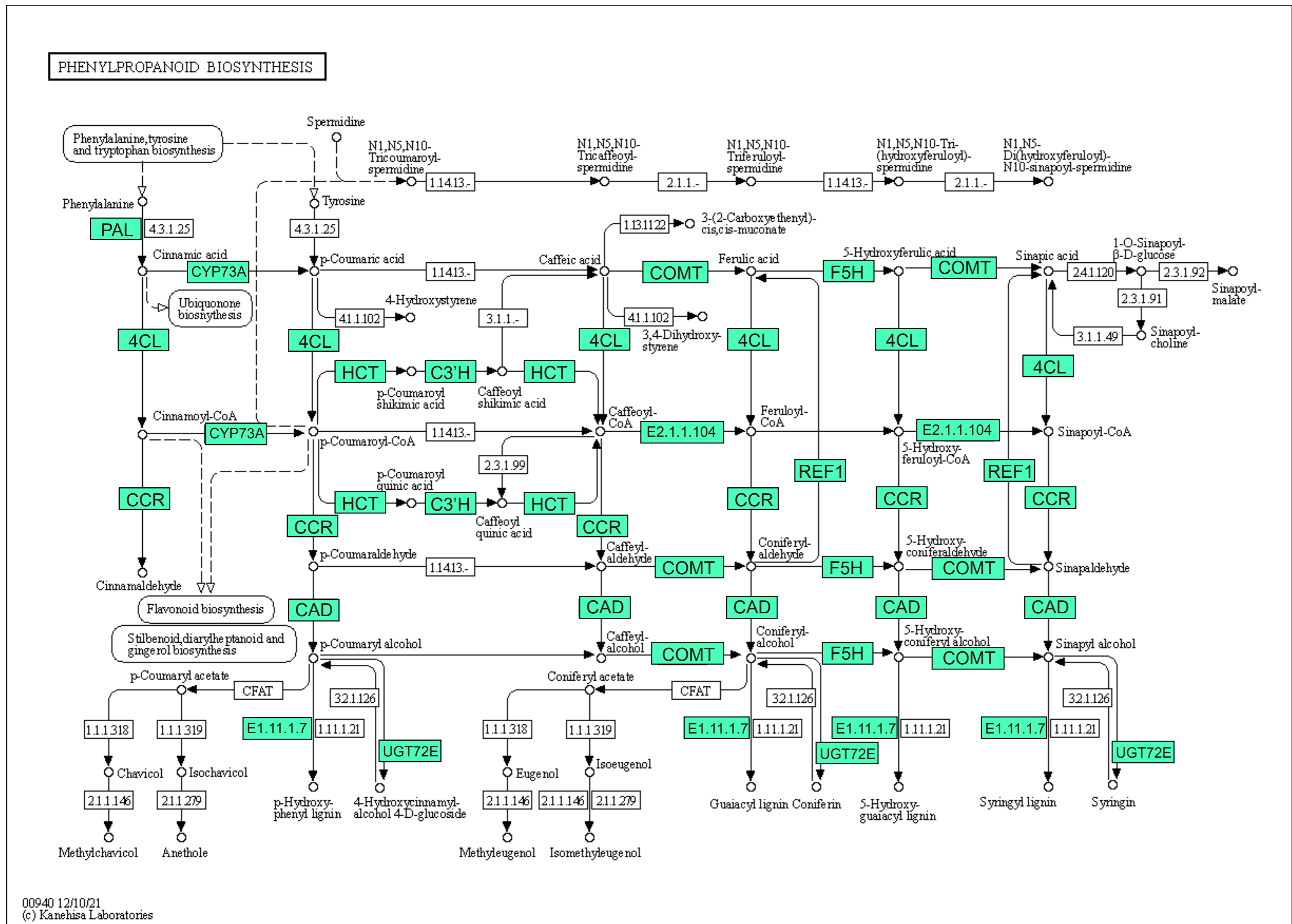**b**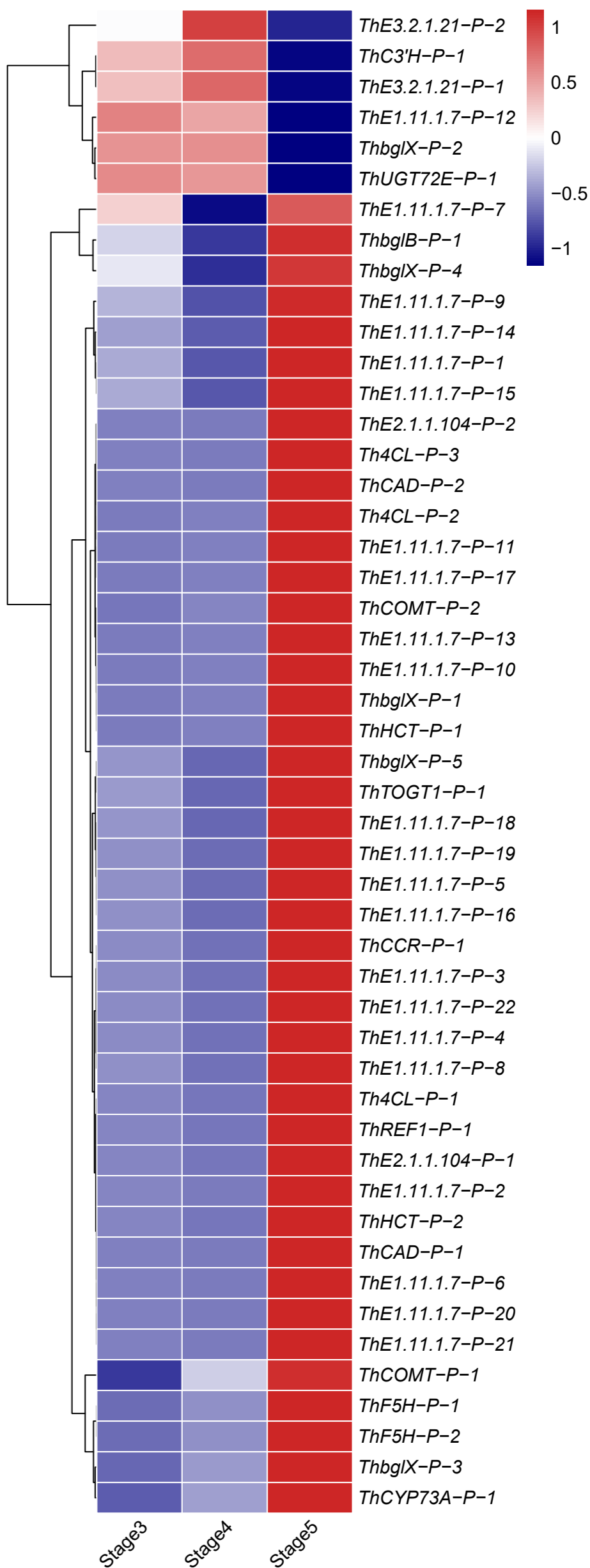

Supplement: Supplementary file 1 [file genes-14-00656-s001.zip › Figure S4.pdf]

PLANT HORMONE SIGNAL TRANSDUCTION

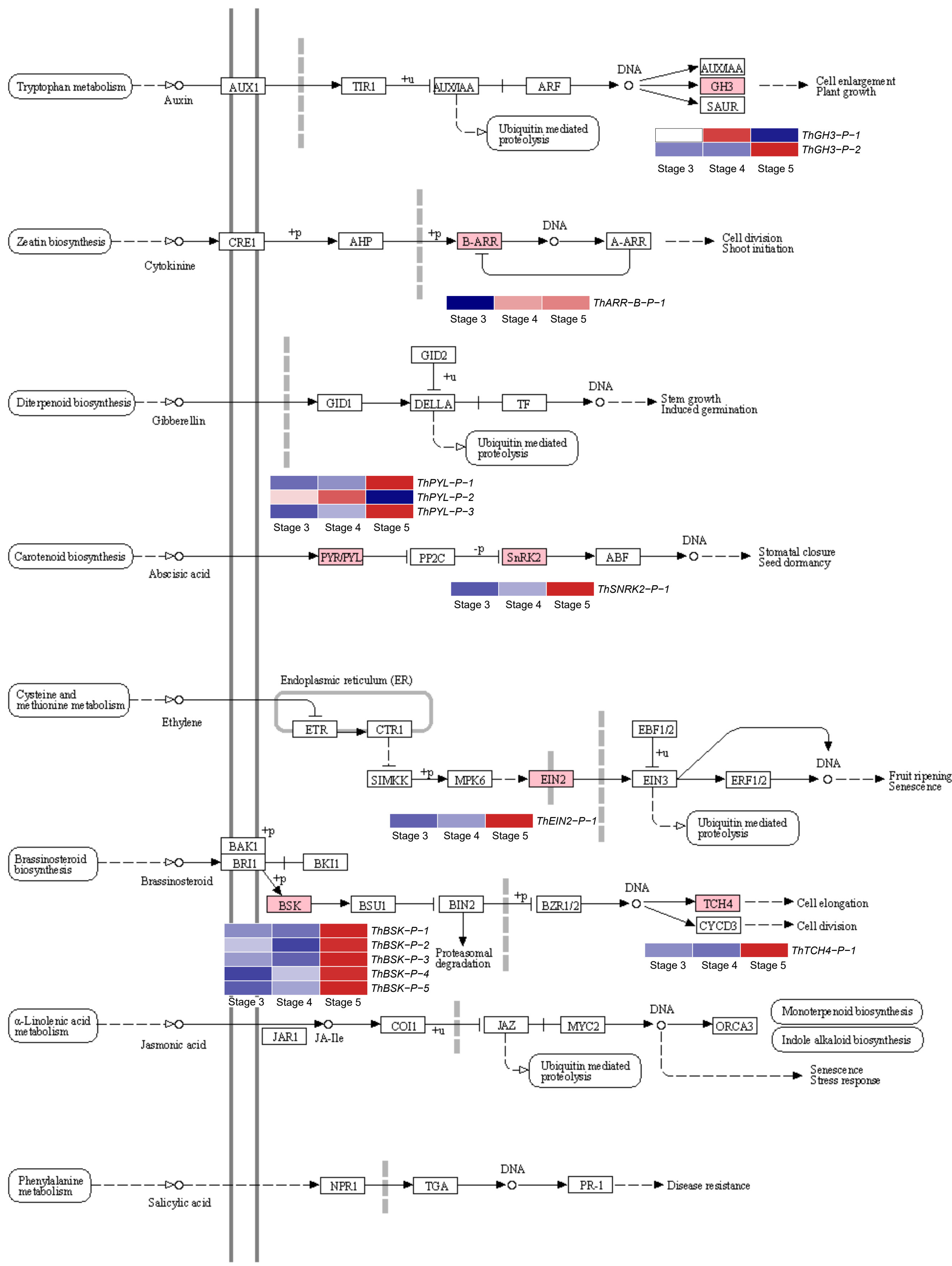

Supplement: Supplementary file 1 [file genes-14-00656-s001.zip › Figure S5.pdf]

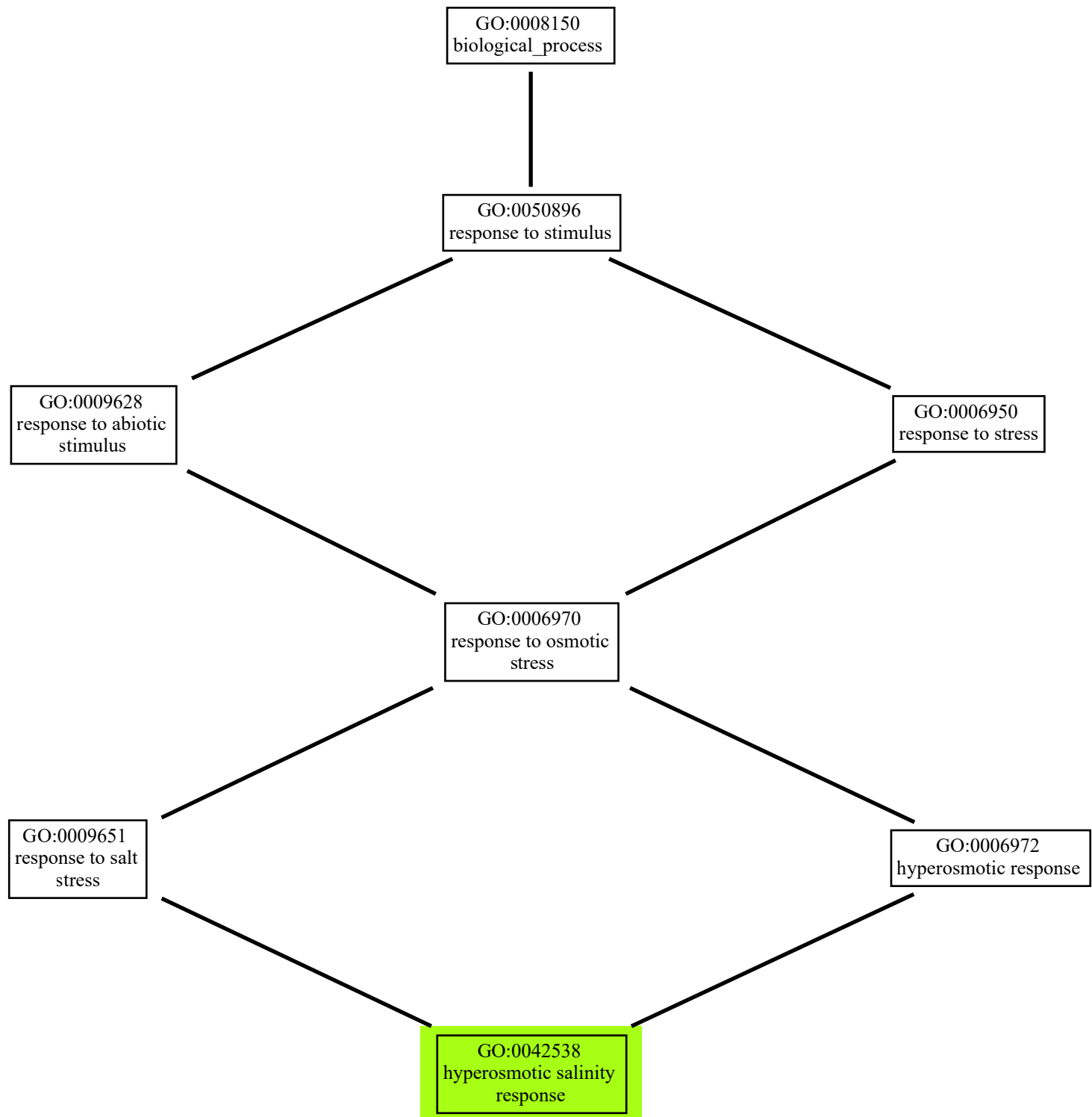

Supplement: Supplementary file 1 [file genes-14-00656-s001.zip › Figure S6.pdf]

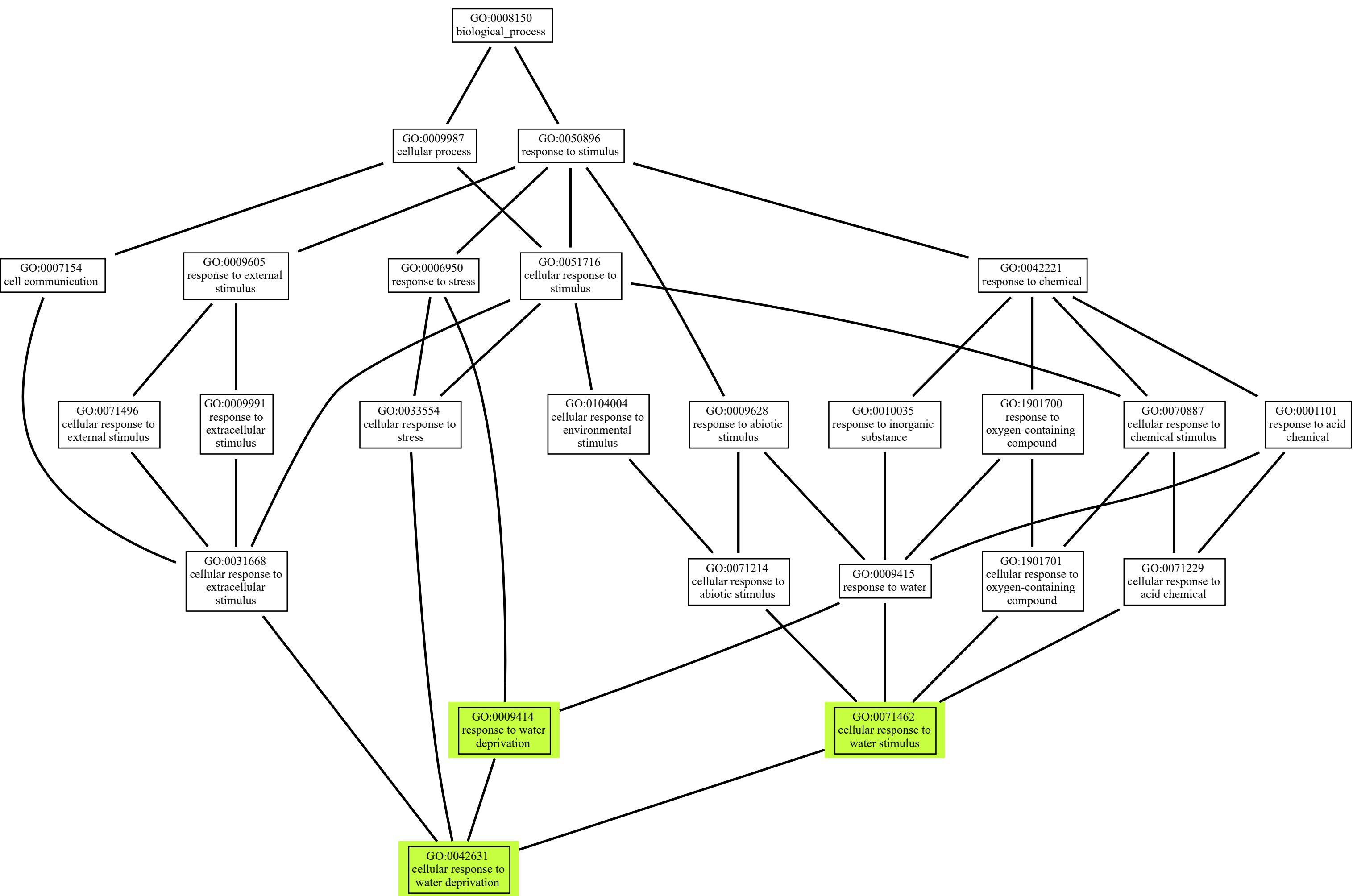

Supplement: Supplementary file 1 [file genes-14-00656-s001.zip › Figure S7.pdf]

a

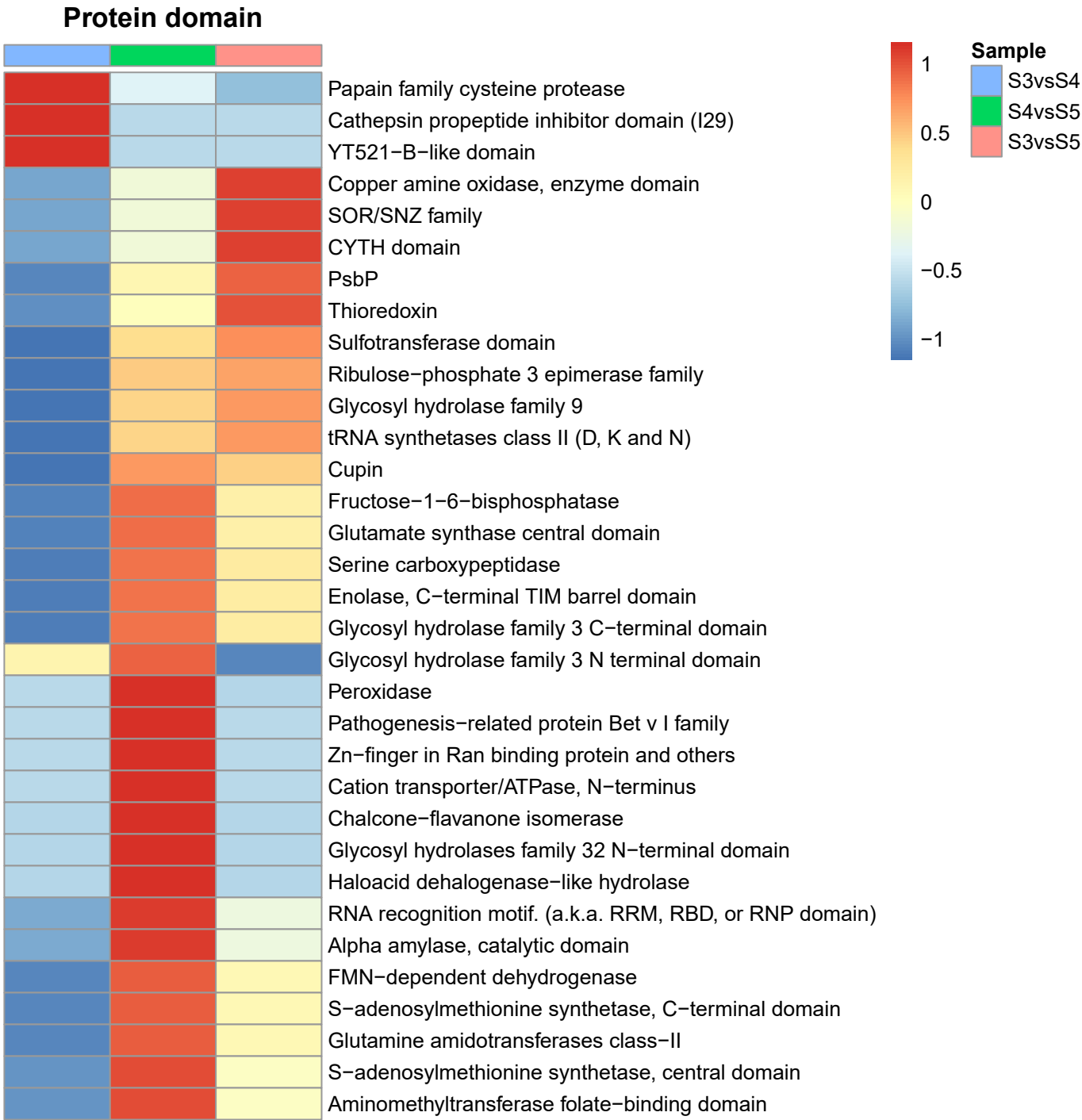

b

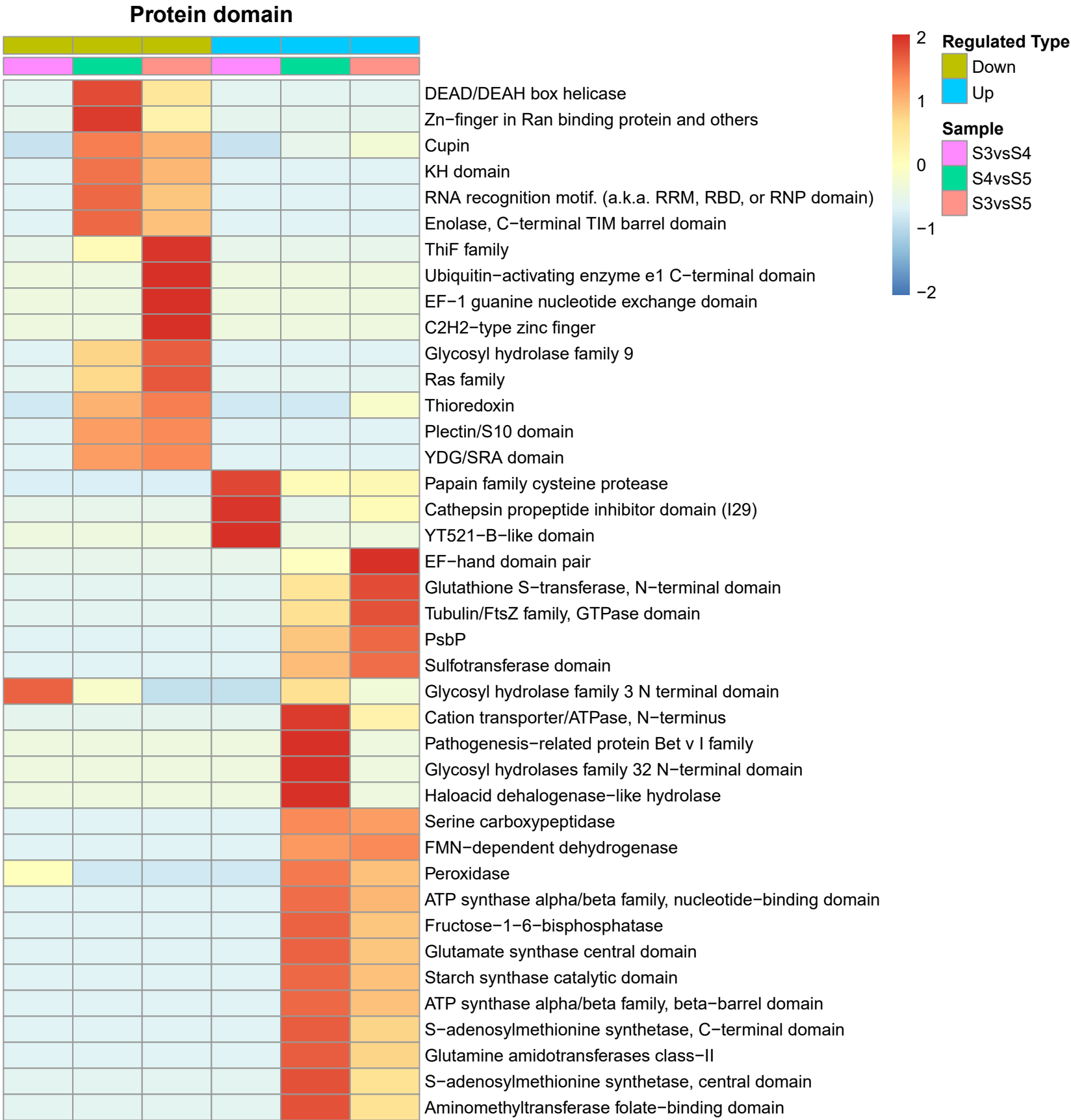

Supplement: Supplementary file 1 [file genes-14-00656-s001.zip › Figure S8.pdf]

a

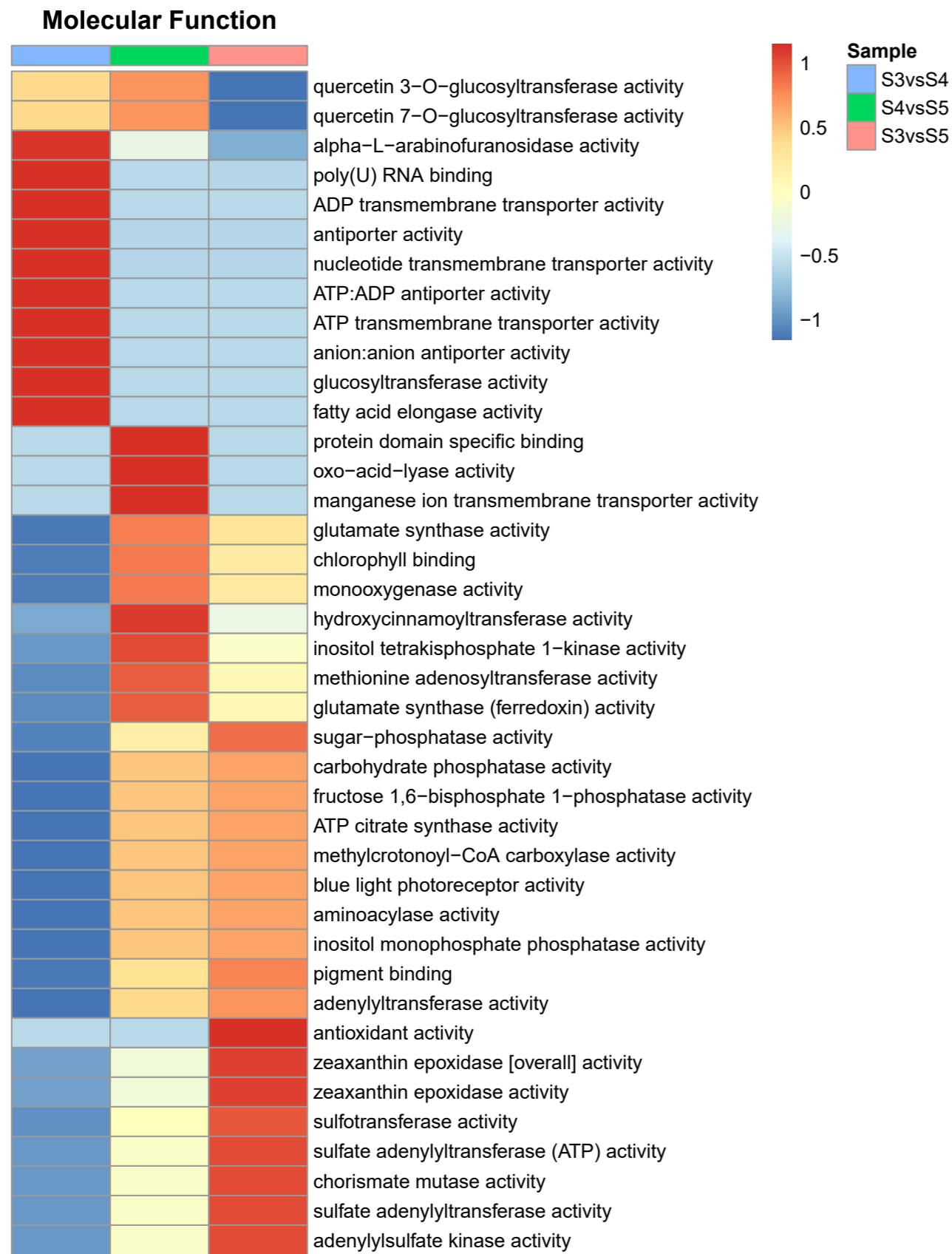

b

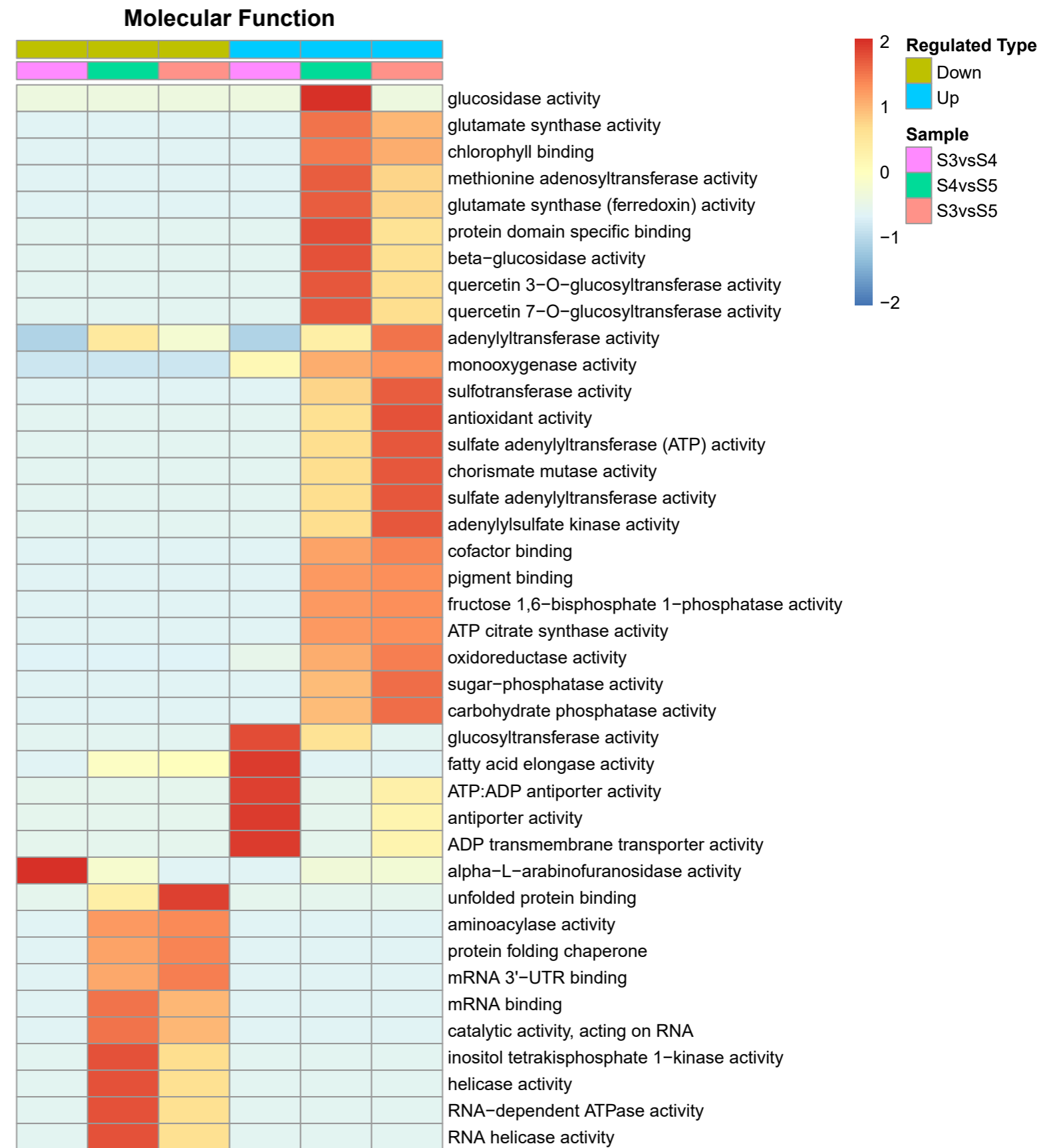

Supplement: Supplementary file 1 [file genes-14-00656-s001.zip › Figure S9.pdf]
